# Difference in the general medicine in-training examination score between community-based hospitals and university hospitals: a cross-sectional study based on 15,188 Japanese resident physicians

**DOI:** 10.1186/s12909-021-02649-0

**Published:** 2021-04-15

**Authors:** Yuji Nishizaki, Keigo Nozawa, Tomohiro Shinozaki, Taro Shimizu, Tomoya Okubo, Yu Yamamoto, Ryota Konishi, Yasuharu Tokuda

**Affiliations:** 1 Medical Technology Innovation Center, Juntendo University, 2-1-1 Hongo, Bunkyo-ku, Tokyo, 113-8421 Japan; 2 Department of Medical Education, Juntendo University School of Medicine, 2-1-1 Hongo, Bunkyo-ku, Tokyo, 113-8421 Japan; 3 Department of Information and Computer Technology, Faculty of Engineering, Tokyo University of Science, 6-3-1 Niijuku, Katsushika-ku, Tokyo, 125-8585 Japan; 4 Department of Diagnostic and Generalist Medicine, Dokkyo Medical University Hospital, 880 Kitakobayashi, Mibumachi, Shimotuga-gun, Tochigi, 321-0293 Japan; 5 Research Division, National Center for University Entrance Examinations, 2-19-23 Komaba, Meguro-ku, Tokyo, 153-8501 Japan; 6 Division of General Medicine, Center for Community Medicine, Jichi Medical University School of Medicine, 3311-1 Yakushiji, Shimotsuke, Tochigi, 329-0498 Japan; 7 Education Adviser Japan Organization of Occupational Health and Safety, 1-1 Kiduki Sumiyoshi-cho, Nakahara-ku, Kawasaki-shi, Kanagawa 211-0021 Japan; 8 General Internal Medicine, Muribushi Okinawa for Teaching Hospitals, 3-42-8 Iso, Urasoe-shi, Okinawa, 901-2132 Japan

**Keywords:** Community-based hospital, Generalist, General medicine in-training examination (GM-ITE), Junior resident physician, University hospital

## Abstract

**Background:**

The general medicine in-training examination (GM-ITE) is designed to objectively evaluate the postgraduate clinical competencies (PGY) 1 and 2 residents in Japan. Although the total GM-ITE scores tended to be lower in PGY-1 and PGY-2 residents in university hospitals than those in community-based hospitals, the most divergent areas of essential clinical competencies have not yet been revealed.

**Methods:**

We conducted a nationwide, multicenter, cross-sectional study in Japan, using the GM-ITE to compare university and community-based hospitals in the four areas of basic clinical knowledge“. Specifically, “medical interview and professionalism,” “symptomatology and clinical reasoning,” “physical examination and clinical procedures,” and “disease knowledge” were assessed.

**Results:**

We found no significant difference in “medical interview and professionalism” scores between the community-based and university hospital residents. However, significant differences were found in the remaining three areas. A 1.28-point difference (95% confidence interval: 0.96–1.59) in “physical examination and clinical procedures” in PGY-1 residents was found; this area alone accounts for approximately half of the difference in total score.

**Conclusions:**

The standardization of junior residency programs and the general clinical education programs in Japan should be promoted and will improve the overall training that our residents receive. This is especially needed in categories where university hospitals have low scores, such as “physical examination and clinical procedures.”

**Supplementary Information:**

The online version contains supplementary material available at 10.1186/s12909-021-02649-0.

## Background

In 1968, the ability to participate in training without medical license after graduation from medical school was abolished and replaced by the clinical training system in Japan, which required physicians to undergo a further ≥2 years of clinical training, even after obtaining their medical license. In 2004, a new residency system was established, making it obligatory for physicians to undergo ≥2 years of residency with a super-rotation curriculum [1]. In Japan, physicians who are in postgraduate clinical training years (PGY) 1 and 2 under this policy are called junior residents.

Although junior residency has been made required since April 2004, the design and operation of these training programs are largely left to the discretion of individual teaching hospitals. No objective outcome measures of clinical training have been established, and the content of the education provided varies between institutions. This lack of standardization results in great disparities in the competency of junior residents [2]. The Japanese Institute for Advancement of Medical Education Program (JAMEP) has aimed to resolve these issues and support residency education [3, 4].

The JAMEP is a non-profit organization established in 2005 to enrich Japanese health care through the support of resident education and thereby improve the quality of medical care [2]. Its main activities are the following: (1) hosting continued professional development lectures and inviting a wide range of physicians, including residents, supervising physicians, and practitioners; (2) providing Web-based platforms where physicians can share their clinical skills, knowledge, and experiences; and (3) conducting the general medicine in-training examination (GM-ITE) for junior residents [3].

The GM-ITE is designed to evaluate the clinical competence of junior residents objectively. The examination was developed by the JAMEP [5]. In line with the early residency objectives of the Japanese Ministry of Health, Labour, and Welfare, the GM-ITE assesses the four areas of basic clinical knowledge, including “medical interview and professionalism,” “symptomatology and clinical reasoning” “physical examination and clinical procedures,” and “disease knowledge.” This comprehensive test covers all disciplines, with a focus on primary care [6].

The purpose of this investigation was to compare GM-ITE score evaluations between the university and community-based hospitals, which are characterized by vast differences in clinical training environments. University hospitals provide more advanced medicine in highly specialized areas than community-based hospitals. Analyzing how these differences impact GM-ITE scores may help to elucidate the distinct training challenges faced by each type of teaching hospital.

We have previously demonstrated that the mean GM-ITE scores of junior residents in university hospitals tend to be lower than those of community-based hospitals [7]. However, the difference in score was centered only on the total GM-ITE scores. As mentioned earlier, because the four distinct domains in the GM-ITE are designed to measure different aspects of clinical competence, the trends in scores attributed to the training environment described could vary depending on the assessed area. In the present investigation, we assessed the reasons underlying the differences in test scores between university hospitals and community-based hospitals.

## Methods

### Study design

This was a nationwide, multicenter, cross-sectional study performed in Japan. We used GM-ITE scores to compare university and community-based hospital residents. This study followed STROBE guidelines.

### Study population

The study population included 15,188 junior residents working in 815 medical institutions nationwide who took the GM-ITE for the first time in the three years from 2016 to 2018. Residents with missing data from the clinical training environment survey questionnaire or examinee characteristics were excluded from the analysis. Data regarding PGY-1 and PGY-2 were analyzed separately because the differences in-training rotations could significantly influence the results.

### General medicine in-training examination

The GM-ITE was developed by the JAMEP in 2011 and serves as an objective evaluation of the essential clinical competencies of junior resident physicians [8–10]. The GM-ITE score consists of the following four categories: “medical interview and professionalism,” “symptomatology and clinical reasoning,” “physical examination and clinical procedures,” and “disease knowledge.” The GM-ITE is a multiple-choice knowledge test and used mainly to assess the experience gained during clinical training. The GM-ITE consisted of 100 multiple choice questions in 2016 and was shortened to 60 questions in 2017 and 2018. The test items cover the following fields: internal medicine, surgery, emergency medicine, pediatrics, obstetrics and gynecology, and psychiatry. After completing the test, resident physicians who took the GM-ITE receive feedback based on the relative scoring of all participants and an educational explanation about each question. This examination is conducted at the end of the year for PGY-1 and PGY-2 junior resident physicians. In line with the early residency objectives of the Japanese Ministry of Health, Labour, and Welfare, the GM-ITE covers the four areas mentioned above of basic clinical knowledge [6]. Thus, the test content and construct validity have been well-established.

The test questions are created through multiple processes over a year. First, all test questions are prepared by a question-creating committee consisting of 22 experienced physicians. Next, all prepared test questions are revised by a peer review committee consisting of five instructors [11].

**Fig. 1:**
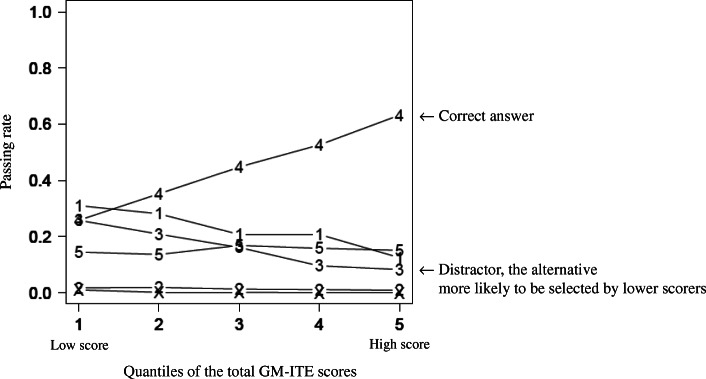
The result of the test analysis for the previous GM-ITE in the field of “disease knowledge.” The vertical axis is the passing rate, and the horizontal axis is the quintile of the total GM-ITE score. The numbers in the figure are the answer choices of the examinees, with “4” as the correct answer

We also performed an analysis on all test questions used in the previous year to improve the quality of the GM-ITE. Figure 1 shows the test analysis results of the previous test question in the field of “disease knowledge.” The test question’s passing rate was 44.5%, and this test question was judged to be a good question as a result of the test analysis because of the effect of a distractor. The correct answer of “4” exhibits a positive correlation with the overall examinee score, while the alternative “3” functions as a distractor (the alternative more likely to be selected by lower scorers). The contents of the test question and detailed explanation of the test analysis are shown in Additional file 1: Appendix 1.

The main purpose of the GM-ITE is to determine the ranking of clinical training facilities’ own grade in Japan and to help overcome their weaknesses in clinical fields. From the point of view of each resident physician, the purpose of the GM-ITE is not to make a pass/fail judgment but to determine the ranking of the resident physician’s own grades in Japan and help overcome their weaknesses in clinical fields. Participating resident physicians will receive detailed medical explanations about the exam questions after the examination. They will also receive feedback about their relative performance in each category (“medical interview and professionalism,” “symptomatology and clinical reasoning,” “physical examination and clinical procedures,” and “disease knowledge”) and each clinical field (internal medicine, surgery, emergency medicine, pediatrics, obstetrics and gynecology, and psychiatry) and the proportion of correct responses. Residency program directors will be informed of the average total scores and proportion of correct responses of the participating residents in their program.

### Data collection

We collected information regarding the clinical training environment from a self-reported questionnaire administered just after the residents completed the GM-ITE. It included GM rotation, emergency department duties per month, the average number of inpatients in charge, use of online medical resources, and study time per day. Hospital information (hospital type [university or community based] and location) was obtained from the Residency Electronic Information System website [12] and the Foundation for the Promotion of Medical Training website [13]. We classified university branch hospitals into university hospitals. Regarding the categories of hospital location, 20 cities designated by the Ministry of Internal Affairs and Communications and the 23 wards in Tokyo were defined as urban cities; and the rest as provincial cities.

### Statistical analyses

GM-ITE total scores were compared between the university and community-based hospitals. We estimated the two-level linear model, adjusting for the hospital- and resident-level variables listed in the Data Collection section and each hospital as an average random intercept. The year of the GM-ITE was also adjusted to account for the difference in maximum scores (i.e., 100 in 2016 and 60 in 2017/2018). Each resident’s GM-ITE total score was divided into four question fields (subcategories); hence, we also modeled the scores at distinct subcategories (i.e., four observations per resident) using the three-level linear model, including hospital- and resident-level random intercepts with subcategory-level error terms. We adjusted for the same variables as the two-level linear model and the dummy variables for subcategories and their product terms with the university/community-based hospital type. All analyses were conducted in PGY-1 and PGY-2, using restricted (for the two-level model) and unrestricted (the three-level model) maximum likelihood methods using the Proc Mixed assessment in SAS ver. 9.4 (Cary, NC).

## Results

The total number of individuals who met the eligibility criteria was 15,188, including 7552 PGY-1 and 7636 PGY-2 resident physicians. After excluding those with missing data, we finally analyzed the data from 7111 PGY-1 and 7154 PGY-2 resident physicians. Table 1 shows that the residents from university hospitals had less experience with emergency department duties, were in charge of fewer inpatients, spent less time studying, and had less experience with GM rotation despite having GM departments.

**Table 1:** Background characteristics of the residents

	PGY-1 residents		PGY-2 residents	
	University hospital	Community-based hospital	University hospital	Community-based hospital
	*n* = 752	*n* = 6359	*n* = 804	*n* = 6350
**Hospital-level variable**				
Hospital Location				
Urban	112 (14.9)	1808 (28.4)	101 (12.6)	1851 (29.1)
Rural	585 (77.8)	4410 (69.4)	671 (83.5)	4382 (69.0)
Other (> 50 million)	55 (7.3)	141 (2.2)	32 (4.0)	117 (1.8)
**Resident-level variable**				
Sex				
Male	479 (63.7)	4316 (67.9)	516 (64.2)	4386 (69.1)
Female	273 (36.3)	2043 (32.1)	288 (35.8)	1964 (30.9)
Emergency department duty per month				
0	105 (14.0)	163 (2.6)	58 (7.2)	120 (1.9)
1 to 2 h	348 (46.3)	698 (11.0)	289 (35.9)	624 (9.8)
3 to 5 h	206 (27.4)	4716 (74.2)	393 (48.9)	4582 (72.2)
> 6 h per month	72 (9.6)	748 (11.8)	45 (5.6)	991 (15.6)
Unknown	21 (2.8)	34 (0.5)	19 (2.4)	33 (0.5)
Average number of inpatients each resident is in charged of				
0 to 4	158 (21.0)	1078 (17.0)	189 (23.5)	868 (13.7)
5 to 9	511 (68.0)	3816 (60.0)	519 (64.6)	3702 (58.3)
10 to 14	69 (9.2)	961 (15.1)	63 (7.8)	1141 (18.0)
> 15	7 (0.9)	287 (4.5)	12 (1.5)	430 (6.8)
Unknown	7 (0.9)	217 (3.4)	21 (2.6)	209 (3.3)
Study time				
0 to 30 min	361 (48.0)	2396 (37.7)	324 (40.3)	2088 (32.9)
31 to 60 min	245 (32.6)	2507 (39.4)	296 (36.8)	2614 (41.2)
61 to 90 min	54 (7.2)	866 (13.6)	108 (13.4)	994 (15.7)
> 91 min	12 (1.6)	242 (3.8)	26 (3.2)	301 (4.7)
None	80 (10.6)	348 (5.5)	50 (6.2)	353 (5.6)
General medicine department rotation				
Yes	186 (24.7)	2119 (33.3)	319 (39.7)	2799 (44.1)
No (in hospitals with a GM department)	552 (73.4)	2775 (43.6)	466 (58.0)	2180 (34.3)
No GM department	14 (1.9)	1465 (23.0)	19 (2.4)	1371 (21.6)
Use of medical online resources				
Unknown/none	287 (38.2)	2736 (43.0)	362 (45.0)	2649 (41.7)
Up-to-date	82 (10.9)	1269 (20.0)	103 (12.8)	1373 (21.6)
Others	383 (50.9)	2354 (37.0)	339 (42.2)	2328 (36.7)
Year				
2016	287 (38.2)	1790 (28.1)	225 (28.0)	1884 (29.7)
2017	239 (31.8)	2144 (33.7)	284 (35.3)	2214 (34.9)
2018	226 (30.1)	2425 (38.1)	295 (36.7)	2252 (35.5)

Data are presented as n (%). PGY indicates postgraduate year

Table 2 presents the average GM-ITE scores of the PGY-1 and PGY-2 resident physicians and shows no areas in which junior residents working in university hospitals scored better than those working in community-based hospitals. The slight fluctuation in point rates (average scores divided by maximum score) between the years suggested that the level of evaluation difficulty is stable.

**Table 2:** Mean (standard deviation) GM-ITE scores of the university and community-based hospital residents

Year	Subcategory	PGY-1		PGY-2	
		University hospitals	Community-based hospitals	University hospitals	Community-based hospitals
2016		*n* = 287	*n* = 1790	*n* = 225	*n* = 1844
	Medical interview and professionalism	10.20 (2.80)	11.77 (2.33)	11.23 (2.57)	11.77 (2.45)
	Symptomatology and clinical reasoning	13.57 (2.66)	14.72 (2.74)	14.15 (2.98)	15.30 (2.81)
	Physical examination and clinical procedures	10.33 (2.70)	12.03 (2.47)	11.46 (2.52)	12.19 (2.64)
	Disease knowledge	14.24 (3.20)	15.73 (3.04)	15.56 (3.49)	16.86 (3.24)
	**Total score (maximum score 100)**	48.34 (8.22)	54.26 (7.30)	52.40 (8.43)	56.13 (8.15)
2017		*n* = 239	*n* = 2144	*n* = 284	*n* = 2214
	Medical interview and professionalism	2.98 (1.02)	3.07 (10.8)	2.87 (1.05)	2.98 (1.12)
	Symptomatology and clinical reasoning	9.01 (2.40)	10.01 (2.39)	9.53 (2.57)	10.43 (2.49)
	Physical examination and clinical procedures	9.46 (2.07)	10.43 (2.20)	9.73 (2.30)	10.52 (2.23)
	Disease knowledge	8.44 (1.86)	9.11 (1.98)	8.18 (2.09)	9.02 (2.10)
	**Total score (maximum score 60)**	29.90 (4.67)	32.62 (5.09)	30.31 (5.43)	32.95 (5.43)
2018		*n* = 226	*n* = 2425	*n* = 295	*n* = 2252
	Medical interview and professionalism	2.82 (1.01)	2.88 (2.30)	2.92 (1.17)	3.09 (1.10)
	Symptomatology and clinical reasoning	9.37 (2.16)	10.01 (2.12)	9.87 (2.08)	10.42 (2.19)
	Physical examination and clinical procedures	8.35 (2.40)	9.43 (2.27)	8.82 (2.26)	9.67 (2.42)
	Disease knowledge	7.49 (2.40)	8.27 (2.30)	8.20 (2.32)	8.79 (2.52)
	**Total score (maximum score 60)**	28.00 (5.59)	30.59 (5.36)	29.81 (5.40)	31.97 (6.00)

GM-ITE indicates general medicine in-training examination; PGY, postgraduate year

Table 3 shows the difference in total GM-ITE scores between the university and community-based hospitals. Additional file 1: Appendix Table 1 presents all the parameter estimates of two-level models. The mean total GM-ITE scores of the PGY-1 and PGY-2 resident physicians were estimated to be 2.52 points (95% confidence interval [CI]: 1.42–3.61) and 1.89 points higher (95% CI: 0.75–3.03) in the community-based hospitals than in the university hospitals, respectively. The differences in mean scores (approximately 2 to 2.5) were similar with the standard deviation of hospital-level variation as follows: the estimates of the standard deviations of hospital-specific random effects were 2.1 = √4.46 (for PGY-1) and 2.4 = √5.61 (for PGY-2; Additional file 1: Appendix Table 1; likewise, the standard deviation of the scores of the residents in each hospital was estimated to range from 5.5 to 6 points from the residual estimates). The scores also positively correlated with study time and GM rotation, as well as several emergency department duties and the average number of inpatients of whom PGY-2 resident physicians were primarily in charge (Additional file 1: Appendix Table 2).

**Table 3:** Estimated differences in GM-ITE total score and scores in the 4 subcategories (university hospitals vs. community-based hospitals)

	PGY-1				PGY-2			
	Difference	95% CI		*P*	Difference	95% CI		*P*
**Two-level model**								
Total score	−2.52	−3.61	−1.42	<.0001	−1.89	−3.0317	−0.7499	0.0012
**Three-level model**								
Medical interview and professionalism	0.00	−0.31	0.31	0.9843	−0.08	−0.4037	0.2373	0.6108
Symptomatology and clinical reasoning	−0.73	−1.04	−0.42	<.0001	−0.60	−0.9252	−0.2842	0.0002
Physical examination and clinical procedures	−1.28	− 1.59	−0.96	<.0001	−0.51	−0.8317	−0.1907	0.0018
Disease knowledge	−0.52	−0.83	−0.21	0.0011	−0.69	−1.0118	−0.3708	<.0001

CI indicates confidence interval; GM-ITE, general medicine in-training examination; PGY, postgraduate year

Table 3 shows the differences in subcategory-specific scores, which were calculated by combining the parameter estimates of three-level models (Additional file 1: Appendix Table 2). No significant difference in “medical interview and professionalism” scores were found between the community-based and university hospital residents. However, significant differences were found for the three remaining areas. A 1.28-point difference (95% CI: 0.96–1.59) in “physical examination and clinical procedures” was found in PGY-1; this area alone accounts for approximately half of the difference in total scores. The difference in this area was also found in the PGY-2 resident physicians. However, the estimated value for the difference was halved to 0.51 points (95% CI: 0.19–0.83), reaching a similar magnitude as did “symptomatology and clinical reasoning” and “disease knowledge.”

## Discussion

This investigation is the first to utilize JAMEP GM-ITE data to formally evaluate the subcategory scores in addition to the total score by using novel multilevel models with statistical interactions. The present study used a three-level model analysis to illustrate the differences in total GM-ITE scores and scores in four subcategories in junior resident physicians according to hospital type. University hospitals had lower scores in all the subcategories when compared to community-based hospitals. We found more significant gaps in the GM-ITE scores for “physical examination and clinical procedures” and negligible gaps in the GM-ITE scores for “medical interview and professionalism.”

As previously mentioned, the Japanese Ministry of Health, Labour, and Welfare introduced a mandatory 2-year postgraduate training program for graduating medical students with a super-rotation curriculum in 2004. Simultaneously, a national matching system was established to determine the hospital residency programs best suited for medical students [1]. Japan has approximately 1000 teaching hospitals and 1300 junior residency programs, including both community-based and university hospitals. This matching system matches candidates with postgraduate clinical training with teaching hospitals that conduct junior residency programs. The matching of a candidate to a hospital is determined by a computer according to an algorithm based on the preferences of both the candidate and the hospital [14, 15]. As a result of the matching data analysis, community-based hospitals tend to be more popular than university hospitals among medical students in the final (sixth) year [15].

In addition to providing advanced medical care, university hospitals also conduct medical research, including essential, translational, and clinical studies. In comparison, community-based hospitals play a more crucial role in treating neighborhoods afflicted with many prevalent diseases. Thus, community-based hospitals are responsible for providing care to as many patients with common diseases as possible. This is presumably why junior resident physicians working in community-based hospitals scored higher in the GM-ITE, which asks questions based on clinical case scenarios that simulate actual situations.

Issues have been raised in association with the education for junior resident physicians of the university and university-affiliated hospitals in Japan. For advancing medical care, advancing the differentiation of specialties of each department was prioritized in university hospitals [16]. Subspecialization may be efficient in terms of promoting academic advances. However, from the perspective of a generalist who treats patients with a wide range of common diseases, the inability to diagnose and treat illnesses outside one’s specialty is highly disadvantageous. One of the main goals for junior residency is learning diagnostic and treatment skills through appropriate clinical reasoning processes that note common symptoms and patients’ conditions in a primary care setting [1]. General physicians who acquire a broad spectrum of essential clinical competencies such as clinical ethics, physical examination, clinical procedures, clinical reasoning, and professionalism play a critical role in the education of junior resident physicians [17, 18].

The three areas with differences in scores, namely, “symptomatology and clinical reasoning,” “physical examination and clinical procedures,” and “disease knowledge,” are all areas in which knowledge and experience can be mainly gained by examining patients at the bedside. “Physical examination and clinical procedures,” in which the difference in scores was wide in PGY-1, is a special area from the point of view of education for resident physicians. That is because it cannot be acquired only by accumulating knowledge. To acquire the sufficient basic clinical skills of physical examination and clinical procedures, senior doctors need to provide consistent education to resident physicians through bedside learning. We inferred that junior resident physicians in university hospitals could have fewer opportunities for systematic bedside learning by senior doctors than those in community-based hospitals.

The number of supervising physicians per junior resident physician is lower in community-based hospitals than in university hospitals, which suggests that junior resident physicians can care for many patients under the same supervising physicians. Junior resident physicians in community-based hospitals are therefore ensured more opportunities for coherent bedside guidance when compared to their colleagues at university hospitals. We think these factors may cause the difference in “physical examination and clinical procedures” scores between the junior resident physicians in university and community-based hospitals.

Physical examinations are essential to the diagnostic approach of both resident physicians and physicians. However, a previous report has indicated that the physical examination skills of primary care physicians have a crucial drawback [19]. In addition, the physical examination skills of resident physicians have been shown to be deteriorating [20]. Moreover, self-confidence in performing physical examinations does not necessarily increase at each stage of training [21]. To improve physical examination teaching skills, a systematic and innovative education program must be developed to hone physical examination skills based on the preceding pedagogy [22, 23].

Physical examination scores were especially low in university hospital programs, and the opportunity to teach these skills could be enhanced by taking advantage of a large number of supervising physicians. In addition, because university hospitals have several specialty departments, they can offer various educational programs in physical examinations. Another alternative suggestion is to organize a comprehensive educational program on physical examinations and procedures for junior residents in university hospital training programs in which the involvement of all departments is mandatory and to require junior residents to participate in the training program. Consequently, we can expect that the performance of this GM-ITE section will improve among university hospital programs.

The results of our study showed that resident physicians in university hospitals spent a shorter amount of time studying. From the perspective of clinical training in junior residency, it is possible that less-motivated resident physicians may have chosen hospitals with shorter duty hours [24]. The number of resident physicians in university hospitals tends to be greater than in community-based hospitals, so the workload of each resident physician in a university hospital could be shorter than that of a resident physician in a community-based hospital. Moreover, I previously revealed that the amount of study time significantly associated with the GM-ITE score [7]. Several previous studies have also indicated the relationship between sufficient self-study time and professional development [25, 26].

We believe that the generalizability of the whole results of this study is not guaranteed. This is because it is expected that each country will have different medical education systems and different roles between university hospitals and community hospitals. However, we believe that the results of this study have provided medical educators around the world with some important insights. First, the GM-ITE score of PGY-1 in the field of “physical examination and clinical procedures” in university hospitals is extremely low. Attention should be paid to the fact that university hospitals tend to adopt an organ-specific educational system. Immediately after the start of junior residency, supervisors will need to see patients at the bedside with the resident physicians and provide consistent, thorough physical examination and education on clinical procedure. Second, evaluating the relationship between the in-training examination score and performance of resident physicians, the focus should be on several aspects, such as the four domains we indicated, rather than focus on only the total score.

This study has several limitations. First, the rotation order differs across departments and between individual junior resident physicians. Thus, the scores in the four categories measured may have been impacted by the content received by the residents immediately before they completed the GM-ITE. Second, the overall number of participants in this investigation was limited. More specifically, the total number of PGY-1 and PGY-2 junior resident physicians was approximately 18,000, but not all the junior resident physicians participated in the GM-ITE. In Japan, there were 1363 residency programs among 1020 hospitals (907 community and 113 university hospitals, including affiliated hospitals) [15]. Our study integrated and used three years of GM-ITE data, with data obtained from approximately 500 clinical training facilities each year. In other words, about half of the clinical training facilities in Japan participated in GM-ITE. It is also worth noting that the number of participants in university hospitals was smaller than that of community-based hospitals, which could have resulted in a selection bias.

We did not assess the resident physicians’ baseline clinical skills. Medical school experience differs between participants, which could have impacted the study results. Finally, this investigation also did not evaluate the number of supervising physicians in each teaching hospital and the years of clinical experience of their residents. These factors could affect the GM-ITE scores. Future prospective studies are needed to confirm the results of this investigation.

## Conclusion

In conclusion, the present study focused on differences between community-based and university hospitals using four subcategories tested via the GM-ITE. The discrepancy in the GM-ITE score by subcategory was investigated to identify more significant gaps between the GM-ITE scores for “physical examination and clinical procedures” and negligible gaps in the GM-ITE scores for “medical interview and professionalism.” To standardize the quality of the junior residency program in Japan, clinical education in categories where university hospitals have low scores, such as “physical examination and clinical procedures,”must be promoted.

## Supplementary Information

**Additional file 1 Appendix 1.** An example of a good-quality question and test analysis. **Appendix Table 1.** Parameter estimates of two-level linear models for the GM-ITE total score. **Appendix Table 2.** Parameter estimates of three-level linear models for the GM-ITE subcategory scores

## Data Availability

The corresponding author will respond to inquiries on the data analyses.

## Abbreviations

CI: confidence interval
GM-ITE: general medicine in-training examination
JAMEP: Japan Institute for Advancement of Medical Education Program
PGY: postgraduate year
