# Supplementary material for: Difference in the general medicine in-training examination score between community-based hospitals and university hospitals: a cross-sectional study based on 15,188 Japanese resident physicians
Source: BMC Med Educ. 2021 Apr 15;21:214. doi: 10.1186/s12909-021-02649-0 (PMC8050907; doi:10.1186/s12909-021-02649-0)
Supplement: Supplementary file 1 — Additional file 1 Appendix 1. An example of a good-quality question and test analysis. Appendix Table 1. Parameter estimates of two-level linear models for the GM-ITE total score. Appendix Table 2. Parameter estimates of three-level linear models for the GM-ITE subcategory scores [file 12909_2021_2649_MOESM1_ESM.docx]

Supplementary file

Research Article

**Superiority of junior residency at community-based hospitals to that at university hospitals: A cross-sectional study based on 15,188 Japanese resident physicians**

Yuji Nishizaki, MD, MPH, PhD^1, 2^; Keigo Nozawa, BS^3^; Tomohiro Shinozaki, PhD, MPH^3^; Taro Shimizu, MD, PhD, MPH, MBA^4^; Tomoya Okubo, PhD^5^; Yu Yamamoto, MD^6^; Ryota Konishi, MD, MS^7^; Yasuharu Tokuda, MD MPH^8^

^1^ Medical Technology Innovation Center, Juntendo University, Tokyo, Japan

^2^Department of Medical Education, Juntendo University School of Medicine, Tokyo, Japan

^3^Department of Information and Computer Technology, Faculty of Engineering, Tokyo University of Science, Tokyo, Japan

^4^Department of Diagnostic and Generalist Medicine, Dokkyo Medical University Hospital, Tochigi, Japan

^5^Research Division, National Center for University Entrance Examinations, Tokyo, Japan

^6^Division of General Medicine, Center for Community Medicine, Jichi Medical University School of Medicine, Tochigi, Japan

^7^Education Adviser Japan Organization of Occupational Health and Safety, Kanagawa, Japan

^8^General Internal Medicine, Muribushi Okinawa for Teaching Hospitals, Okinawa, Japan

**Institutional addresses:**

^1, 2^2-1-1 Hongo, Bunkyo-ku, Tokyo 113-8421, Japan

^3^6-3-1 Niijuku, Katsushika-ku, Tokyo 125-8585, Japan

^4^880 Kitakobayashi, Mibumachi, Shimotuga-gun, Tochigi 321-0293, Japan

^5^2-19-23 Komaba, Meguro-ku, Tokyo 153-8501, Japan

^6^3311-1 Yakushiji, Shimotsuke, Tochigi 329-0498, Japan

^7^1-1 Kiduki Sumiyoshi-cho, Nakahara-ku, Kawasaki-shi, Kanagawa 211-0021, Japan

^8^3-42-8 Iso, Urasoe-shi, Okinawa 901-2132, Japan

**Corresponding author:** Yuji Nishizaki, MD, MPH, PhD

Medical Technology Innovation Center, Juntendo University, 2-1-1 Hongo, Bunkyo-ku, Tokyo 113-8421, Japan.

Tel: +81-3-3813-3111; Fax: +81-3-5689-0627

E-mail: [ynishiza@juntendo.ac.jp](mailto:ynishiza@juntendo.ac.jp)

**Appendix 1. An example of a good-quality question and test analysis**

After the GM-ITE is completed, all questions will undergo analysis for evaluation of the quality of each question. Test analysis results will be used to improve future questions. An example of the test analysis is shown in Figure 1.

**Question**

The patient, a 60-year-old woman, had been hospitalized after a fall. She was diagnosed as having a left tibial fracture at 1 month prior to the current presentation. She presented with increased pain in her left lower leg. She did not have dyspnea. However, severe swelling was observed in her left lower leg. There was difference in color between the left and right legs, and the skin on the left side of the lower leg was dark red in color. The legs were afebrile. Venous doppler ultrasonography and computed tomography (CT) revealed thrombosis in the popliteal vein. Echocardiography revealed no evidence of right heart overload.

Which is the most appropriate approach?

1. Inferior vena cava (IVC) filter placement
2. Initiate thrombolytic therapy with t-PA
3. Initiate thrombolytic therapy with urokinase
4. Anticoagulant therapy
5. Contrast-enhanced chest CT

**Correct answer**

(4) Anticoagulant therapy

**Summary of Explanation**

Anticoagulant therapy should be initiated. The patient presented changes in skin color that differed between the left and right sides, and edema; furthermore, imaging modalities identified a thrombus in the popliteal vein. These findings and her medical history support that she had deep vein thrombosis (DVT). A temporary IVC filter placement would not be an appropriate response because of the risk of infection. As no right heart overload was present, the patient also is not eligible to undergo thrombolytic therapy with t-PA or urokinase. While catheter-directed thrombolysis with urokinase has been shown to prevent post-thrombotic syndrome, it is usually indicated for DVT in the iliac and femoral vein areas and would not be suitable for the present patient. Although contrast-enhanced chest CT is a routine procedure commonly performed in Japan, it is not a necessary test for this case.[1]

**Evidence of a good-quality question**

You will only be able to arrive at the correct answer for this question if you go through the thought process shown in the following: the first step is “recalling the disease,” followed by “interpreting the state of the disease” and then “decision of management.”

Step 1. Diagnosis

Deep vein thrombosis can be diagnosed on the basis of the keywords “unilateral,” “acute,” “lower leg,” and “pain.”

Step 2. Interpretation (In this question, formulating the distractor which the option more likely to be selected by lower scorers is a key point.)

As no findings were indicative of right heart overload, this can be understood as the patient having no pulmonary embolism requiring thrombolytic therapy or thrombectomy and therefore as a reason that the patient requires treatment for distal deep phlebothrombosis.

Step 3. Management

Therefore, anticoagulant therapy is selected as the appropriate form of management.

This question can be of high quality, as it requires the examinee to go through the three steps mentioned earlier.

**Interpretation of the test analysis results**

The correct response rate (choice “4”) for this question positively correlated with the GM-ITE high score (the quintile of the total GM-ITE score). Moreover choice “3” functions as a distractor (the choice more likely to be selected by lower scorers). The analysis results indicated that examinees with lower scores were more likely to misjudge at the “interpretation” step even if they managed to successfully clear the “diagnosis” step shown earlier. Moreover, they tended to select the distractor (choice “3”) targeting the proximal thrombus. Thrombolytic therapy with urokinase, shown in choice “3,” is somewhat excessive management for the patient described in the question. Junior resident physicians with basic clinical competency can correctly identify it.

**Reference**

1. Ando T, Ito M, Ogi N, Kobayashi T, Tajima H, Nakanishi N, et al.: Guidelines for the Diagnosis and Treatment of Circulatory Diseases (Joint research group 2008 report) [Digest version] Guidelines for the Diagnosis, Treatment and Prevention of Pulmonary Thromboembolism and Deep Vein Thrombosis (2009 revised version), Japanese Journal of Cardiovascular Surgery. 2014;43: 1-21. (in Japanese)

| Appendix Table 1. Parameter estimates of two-level linear models for the GM-ITE total score | | | | | |  |  |  |  |  |
| --- | --- | --- | --- | --- | --- | --- | --- | --- | --- | --- |
|  |  | PGY-1 | | | |  | PGY-2 | | | |
|  |  | Estimate | 95% CI | | *P* |  | Estimate | 95% CI | | *P* |
| **Fixed effect (hospital level)** | |  |  |  |  |  |  |  |  |  |
| Hospital type | |  |  |  |  |  |  |  |  |  |
|  | University | −2.52 | −3.61 | −1.42 | <.0001 |  | −1.89 | −3.03 | −0.75 | 0.0012 |
|  | Community based | Ref | | | |  | Ref | | | |
| Hospital Location | |  |  |  |  |  |  |  |  |  |
|  | Urban | Ref | | | |  | Ref | | | |
|  | Rural | −0.07 | −0.59 | 0.45 | 0.7966 |  | 0.20 | −0.36 | 0.76 | 0.4875 |
|  | Other (>50 million) | −1.21 | −2.70 | 0.28 | 0.1102 |  | −1.90 | −3.68 | −0.11 | 0.0373 |
| **Fixed effect (resident level)** | |  |  |  |  |  |  |  |  |  |
| Sex | |  |  |  |  |  |  |  |  |  |
|  | Male | Ref | | | |  | Ref | | | |
|  | Female | −0.43 | −0.71 | −0.15 | 0.0027 |  | −0.50 | −0.81 | −0.19 | 0.0015 |
| Emergency department duty per month | |  |  |  |  |  |  |  |  |  |
|  | 0 | Ref | | | |  | Ref | | | |
|  | 1 to 2 | 0.03 | −0.78 | 0.84 | 0.9433 |  | 1.39 | 0.36 | 2.42 | 0.008 |
|  | 3 to 5 | 0.64 | −0.11 | 1.39 | 0.096 |  | 2.10 | 1.14 | 3.06 | <.0001 |
|  | >6 | 0.66 | −0.20 | 1.52 | 0.1308 |  | 1.80 | 0.75 | 2.85 | 0.0007 |
|  | Unknown | −1.07 | −2.70 | 0.56 | 0.1987 |  | 2.33 | 0.42 | 4.23 | 0.0166 |
| Average number of inpatients each resident is in charged of | |  |  |  |  |  |  |  |  |  |
|  | 0 to 4 | Ref | | | |  | Ref | | | |
|  | 5 to 9 | 0.45 | 0.06 | 0.83 | 0.0227 |  | 1.32 | 0.88 | 1.76 | <.0001 |
|  | 10 to 14 | 0.60 | 0.09 | 1.11 | 0.0218 |  | 1.33 | 0.78 | 1.89 | <.0001 |
|  | >15 | 0.30 | −0.50 | 1.10 | 0.4629 |  | 1.91 | 1.10 | 2.73 | <.0001 |
|  | Unknown | −0.51 | −1.33 | 0.31 | 0.2223 |  | 0.56 | −0.34 | 1.46 | 0.2261 |
| Study time | |  |  |  |  |  |  |  |  |  |
|  | 0 to 30 min | Ref | | | |  | Ref | | | |
|  | 31 to 60 min | 0.74 | 0.44 | 1.04 | <.0001 |  | 0.59 | 0.26 | 0.93 | 0.0005 |
|  | 61 to 90 min | 0.92 | 0.49 | 1.35 | <.0001 |  | 1.06 | 0.62 | 1.51 | <.0001 |
|  | >91 min | 2.72 | 1.99 | 3.46 | <.0001 |  | 1.95 | 1.23 | 2.67 | <.0001 |
|  | None | −0.63 | −1.20 | −0.06 | 0.0313 |  | −1.27 | −1.92 | −0.62 | 0.0001 |
| General medicine department rotation | |  |  |  |  |  |  |  |  |  |
|  | Yes | Ref | | | |  | Ref | | | |
|  | No (in hospitals with a GM department) | −0.61 | −0.98 | −0.25 | 0.001 |  | −0.31 | −0.70 | 0.07 | 0.1089 |
|  | No GM department | −1.27 | −1.76 | −0.78 | <.0001 |  | −0.65 | −1.18 | −0.12 | 0.0171 |
| Use of medical online resources | |  |  |  |  |  |  |  |  |  |
|  | Unknown/none | Ref | | | |  | Ref | | | |
|  | Up-to-date | 0.79 | 0.41 | 1.17 | <.0001 |  | 0.61 | 0.20 | 1.01 | 0.0037 |
|  | Others | −0.37 | −0.68 | −0.07 | 0.0163 |  | −0.94 | −1.28 | −0.60 | <.0001 |
| Year | |  |  |  |  |  |  |  |  |  |
|  | 2016 | Ref | | | |  | Ref | | | |
|  | 2017 | −21.13 | −21.55 | −20.72 | <.0001 |  | −23.01 | −23.47 | −22.56 | <.0001 |
|  | 2018 | −22.98 | −23.39 | −22.58 | <.0001 |  | −23.93 | −24.38 | −23.48 | <.0001 |
| **Random effect** | |  |  |  |  |  |  |  |  |  |
| Variance of random intercept (between-hospital variation) | | 4.46 |  |  |  |  | 5.61 |  |  |  |
|  | Standard error | 0.47 |  |  |  |  | 0.60 |  |  |  |
| Variance of error (within-hospital variation) | | 29.59 |  |  |  |  | 35.18 |  |  |  |
|  | Standard error | 0.52 |  |  |  |  | 0.62 |  |  |  |
| CI indicates confidence interval; GM-ITE, general medicine in-training examination; PGY, postgraduate year. | | | | | | | |  |  |  |

| Appendix Table 2. Parameter estimates of three-level linear models for the GM-ITE subcategory scores | | | | | | | |  |  |  |
| --- | --- | --- | --- | --- | --- | --- | --- | --- | --- | --- |
|  |  | PGY-1 | | | |  | PGY-2 | | | |
|  |  | Estimate | 95% CI | | *P* |  | Estimate | 95% CI | | *P* |
| **Fixed effect (hospital level)** | |  |  |  |  |  |  |  |  |  |
| Hospital type | |  |  |  |  |  |  |  |  |  |
|  | University | −0.73 | 1.04 | 0.42 | <.0001 |  | −0.60 | −0.93 | −0.28 | 0.0002 |
|  | Community-based | Ref | | | |  | Ref | | | |
| Hospital Location | |  |  |  |  |  |  |  |  |  |
|  | Urban | Ref | | | |  | Ref | | | |
|  | Rural | −0.02 | 0.15 | 0.11 | 0.7932 |  | 0.05 | −0.09 | 0.19 | 0.4853 |
|  | Other (>50 million) | −0.30 | 0.67 | 0.07 | 0.1073 |  | −0.47 | −0.92 | −0.03 | 0.0358 |
| **Fixed effect (resident level)** | |  |  |  |  |  |  |  |  |  |
| Sex | |  |  |  |  |  |  |  |  |  |
|  | Male | Ref | | | |  | Ref | | | |
|  | Female | −0.11 | 0.18 | 0.04 | 0.0026 |  | −0.13 | −0.20 | −0.05 | 0.0014 |
| Emergency department duty per month | |  |  |  |  |  |  |  |  |  |
|  | 0 | Ref | | | |  | Ref | | | |
|  | 1 to 2 | 0.01 | 0.19 | 0.21 | 0.9399 |  | 0.35 | 0.09 | 0.60 | 0.0079 |
|  | 3 to 5 | 0.16 | 0.03 | 0.35 | 0.0938 |  | 0.52 | 0.28 | 0.76 | <.0001 |
|  | >6 | 0.17 | 0.05 | 0.38 | 0.1293 |  | 0.45 | 0.19 | 0.71 | 0.0007 |
|  | Unknown | −0.27 | 0.67 | 0.14 | 0.199 |  | 0.58 | 0.11 | 1.06 | 0.0164 |
| Average number of inpatients each resident is in charged of | |  |  |  |  |  |  |  |  |  |
|  | 0 to 4 | Ref | | | |  | Ref | | | |
|  | 5 to 9 | 0.11 | 0.02 | 0.21 | 0.0222 |  | 0.33 | 0.22 | 0.44 | <.0001 |
|  | 10 to 14 | 0.15 | 0.02 | 0.28 | 0.0214 |  | 0.33 | 0.19 | 0.47 | <.0001 |
|  | >15 | 0.08 | 0.13 | 0.28 | 0.4617 |  | 0.48 | 0.28 | 0.68 | <.0001 |
|  | Unknown | −0.13 | 0.33 | 0.08 | 0.222 |  | 0.14 | −0.09 | 0.36 | 0.226 |
| Study time | |  |  |  |  |  |  |  |  |  |
|  | 0 to 30 min | Ref | | | |  | Ref | | | |
|  | 31 to 60 min | 0.18 | 0.11 | 0.26 | <.0001 |  | 0.15 | 0.06 | 0.23 | 0.0005 |
|  | 61 to 90 min | 0.23 | 0.12 | 0.34 | <.0001 |  | 0.27 | 0.15 | 0.38 | <.0001 |
|  | >91 min | 0.68 | 0.50 | 0.86 | <.0001 |  | 0.49 | 0.31 | 0.67 | <.0001 |
|  | None | −0.16 | 0.30 | 0.01 | 0.031 |  | −0.32 | −0.48 | −0.15 | 0.0001 |
| General medicine department rotation | |  |  |  |  |  |  |  |  |  |
|  | Yes | Ref | | | |  | Ref | | | |
|  | No (in hospitals with a GM department) | −0.15 | 0.24 | 0.06 | 0.0009 |  | −0.08 | −0.18 | 0.02 | 0.1044 |
|  | No GM department | −0.32 | 0.44 | 0.20 | <.0001 |  | −0.16 | −0.29 | −0.03 | 0.0161 |
| Use of medical online resources | |  |  |  |  |  |  |  |  |  |
|  | Unknown/none | Ref | | | |  | Ref | | | |
|  | Up-to-date | 0.20 | 0.10 | 0.29 | <.0001 |  | 0.15 | 0.05 | 0.25 | 0.0035 |
|  | Others | −0.09 | 0.17 | 0.02 | 0.0159 |  | −0.24 | −0.32 | −0.15 | <.0001 |
| Year | |  |  |  |  |  |  |  |  |  |
|  | 2016 | Ref | | | |  | Ref | | | |
|  | 2017 | −5.28 | 5.39 | 5.18 | <.0001 |  | −5.75 | −5.87 | −5.64 | <.0001 |
|  | 2018 | −5.75 | 5.85 | 5.65 | <.0001 |  | −5.98 | −6.09 | −5.87 | <.0001 |
| GM-ITE subcategory | |  |  |  |  |  |  |  |  |  |
|  | Medical interview and professionalism | −5.89 | 5.97 | 5.81 | <.0001 |  | −6.24 | −6.33 | −6.16 | <.0001 |
|  | Symptomatology and clinical reasoning | Ref | | | |  | Ref | | | |
|  | Physical examination and clinical procedures | −0.84 | 0.92 | 0.76 | <.0001 |  | −1.16 | −1.24 | −1.08 | <.0001 |
|  | Disease knowledge | −0.69 | 0.77 | 0.61 | <.0001 |  | −0.61 | −0.69 | −0.53 | <.0001 |
| GM-ITE subcategory * hospital type | |  |  |  |  |  |  |  |  |  |
|  | Medical interview and professionalism * University hospital | 0.73 | 0.48 | 0.98 | <.0001 |  | 0.52 | 0.28 | 0.77 | <.0001 |
|  | Symptomatology and clinical reasoning * University hospital | Ref | | | |  | Ref | | | |
|  | Physical examination and clinical procedures * University hospital | −0.55 | 0.80 | 0.30 | <.0001 |  | 0.09 | −0.15 | 0.34 | 0.4552 |
|  | Disease knowledge * University hospital | 0.21 | 0.04 | 0.45 | 0.0985 |  | −0.09 | −0.33 | 0.16 | 0.489 |
| **Random effect** | |  |  |  |  |  |  |  |  |  |
| Variance of random hospital effect (between-hospital variation) | | 0.27 |  |  |  |  | 0.34 |  |  |  |
|  | Standard error | 0.03 |  |  |  |  | 0.04 |  |  |  |
| Variance of random resident effect (within-hospital and between-resident variation) | | 0.51 |  |  |  |  | 0.80 |  |  |  |
|  | Standard error | 0.03 |  |  |  |  | 0.04 |  |  |  |
| Variance of error (within-resident variation) | | 5.32 |  |  |  |  | 5.59 |  |  |  |
|  | Standard error | 0.05 |  |  |  |  | 0.05 |  |  |  |
| CI indicates confidence interval; GM-ITE, general medicine in-training examination; PGY, postgraduate year. | | | | | | |  |  |  |  |
